# The genomic and bulked segregant analysis of *Curcuma alismatifolia* revealed its diverse bract pigmentation

**DOI:** 10.1007/s42994-022-00081-6

**Published:** 2022-10-06

**Authors:** Xuezhu Liao, Yuanjun Ye, Xiaoni Zhang, Dan Peng, Mengmeng Hou, Gaofei Fu, Jianjun Tan, Jianli Zhao, Rihong Jiang, Yechun Xu, Jinmei Liu, Jinliang Yang, Wusheng Liu, Luke R. Tembrock, Genfa Zhu, Zhiqiang Wu

**Affiliations:** 1 Shenzhen Branch, Guangdong Laboratory of Lingnan Modern Agriculture, Genome Analysis Laboratory of the Ministry of Agriculture and Rural Affairs, Agricultural Genomics Institute at Shenzhen, Chinese Academy of Agricultural Sciences, Shenzhen, 518120 China; 2 Guangdong Provincial Key Lab of Ornamental Plant Germplasm Innovation and Utilization, Environmental Horticulture Research Institute, Guangdong Academy of Agricultural Sciences, Guangzhou, 510640 China; 3 Yunnan Key Laboratory of Plant Reproductive Adaptation and Evolutionary Ecology, Yunnan University, Kunming, 650504 China; 4 Guangxi Engineering and Technology Research Center for Woody Spices, Guangxi Key Laboratory for Cultivation and Utilization of Special Non-Timber Forest Crops, Guangxi Forestry Research Institute, Nanning, 530002 China; 5 Department of Agronomy and Horticulture, University of Nebraska-Lincoln, Lincoln, NE 68583 USA; 6 Department of Horticultural Science, North Carolina State University, Raleigh, NC 27607 USA; 7 Department of Agricultural Biology, Colorado State University, Fort Collins, CO 80523 USA; 8 Kunpeng Institute of Modern Agriculture at Foshan, Foshan, 528200 China

**Keywords:** Anthocyanin synthesis, Siam tulip, Floriculture, Zingiberaceae, Genome evolution

## Abstract

Compared with most flowers where the showy part comprises specialized leaves (petals) directly subtending the reproductive structures, most Zingiberaceae species produce showy “flowers” through modifications of leaves (bracts) subtending the true flowers throughout an inflorescence. *Curcuma alismatifolia*, belonging to the Zingiberaceae family, a plant species originating from Southeast Asia, has become increasingly popular in the flower market worldwide because of its varied and esthetically pleasing bracts produced in different cultivars. Here, we present the chromosome-scale genome assembly of *C. alismatifolia* “Chiang Mai Pink” and explore the underlying mechanisms of bract pigmentation. Comparative genomic analysis revealed *C. alismatifolia* contains a residual signal of whole-genome duplication. Duplicated genes, including pigment-related genes, exhibit functional and structural differentiation resulting in diverse bract colors among *C. alismatifolia* cultivars. In addition, we identified the key genes that produce different colored bracts in *C. alismatifolia*, such as *F3′5'H*, *DFR*, *ANS* and several transcription factors for anthocyanin synthesis, as well as *chlH* and *CAO* in the chlorophyll synthesis pathway by conducting transcriptomic analysis, bulked segregant analysis using both DNA and RNA data, and population genomic analysis. This work provides data for understanding the mechanism of bract pigmentation and will accelerate breeding in developing novel cultivars with richly colored bracts in *C. alismatifolia* and related species. It is also important to understand the variation in the evolution of the Zingiberaceae family.

**Supplementary Information:**

The online version contains supplementary material available at 10.1007/s42994-022-00081-6.

## Introduction

Zingiberaceae is a monocotyledonous angiosperm lineage family that contains several important crops, such as ginger (*Zingiber officinale*), cardamom (*Elettaria cardamomum*), and turmeric (*Curcuma longa*). In addition to the important economic value, the special flower structure and the complex habitat of Zingiberaceae have led to the evolution of many unique reproductive modes and pollination mechanisms (Sun et al. 2011; Wang et al. 2004). Therefore, Zingiberaceae plays an important role in the study of plant phylogeny and evolution of reproductive systems. As the most challenging genus in Zingiberaceae, the classification of *Curcuma* is plagued by polyploid speciation and homoploid hybridization and the division of the genus has always been a controversial issue owing to molecular and morphological conflicts (Záveská et al. 2012). However, *Curcuma* species including *C. alismatifolia* are of great importance because of their long history of use as medicines, including *C. alismatifolia* (Akter et al. 2008; Taheri et al. 2019). In addition to their pharmacological properties, this genus also contains diverse ornamental species with showy bracteate inflorescences they produce. Thus, it is important to decode the underlying genetic basis of these traits in those ornamental species.

**Fig. 1:**
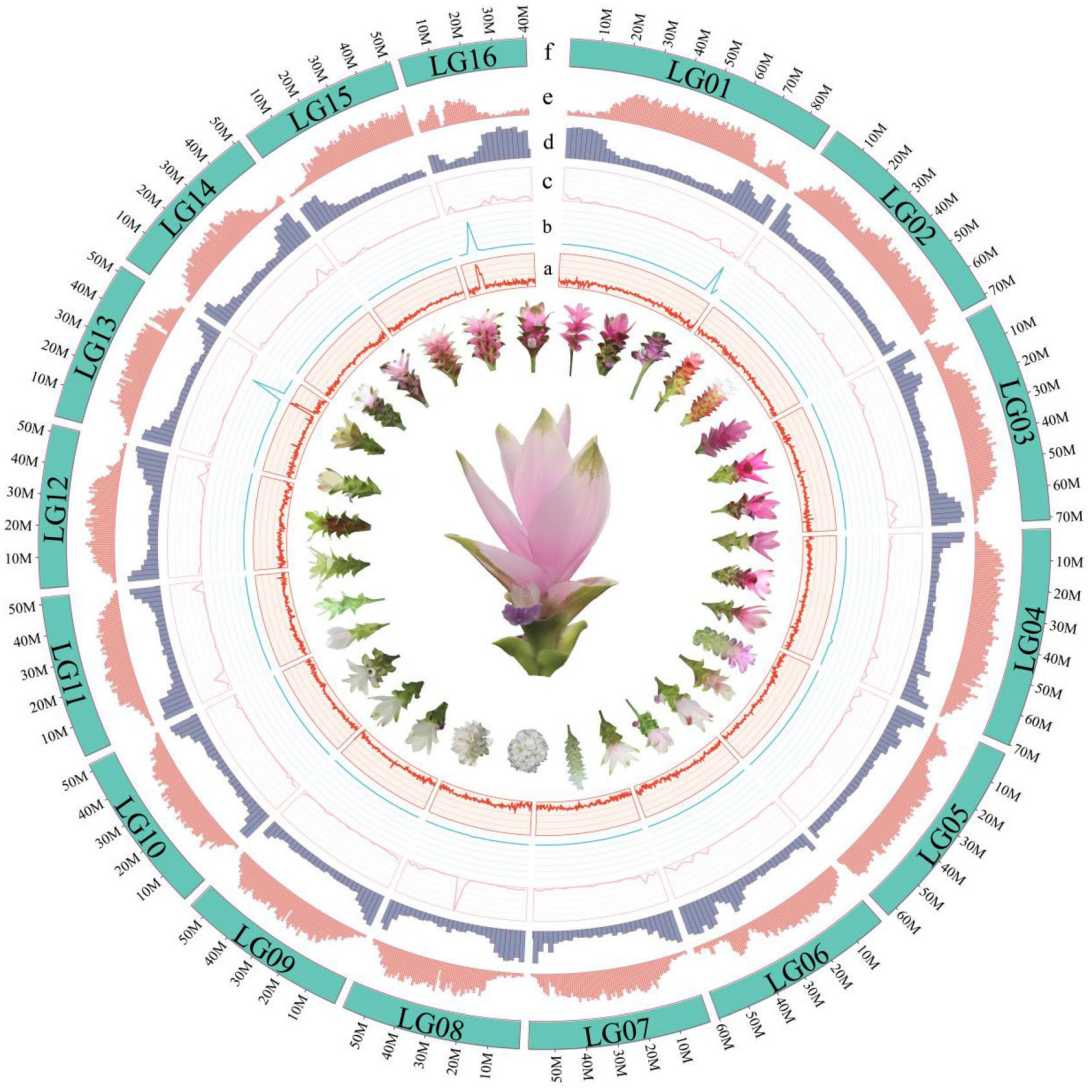
Structure of the *C. alismatifolia* genome. The innermost circle shows the diversity of bract pigmentation and inflorescence morphology. **A** GC content. **B** rRNA distribution. **C** tRNA distribution. **D** SSR distribution. **E** LTR distribution. **F** 16 chromosomes

*Curcuma alismatifolia* Gagnep is a tropical species native to Cambodia, Laos, and Thailand and is commonly known as Siam tulip. Their “flowers” consist of a series of large colorful bract-subtending flowers in a spike inflorescence (Fig. 1). It is a very popular ornamental cut or potted flower in China and Southeast Asia because of its distinctive inflorescence, colorful bracts, and a long flowering period that lasts from May to November during the high-temperature season. This extended period of flowering is particularly attractive among floriculturists, as it provides a longer production window than most cut flowers. Among the many cultivars of Siam tulip, “Chiang Mai Pink” is the most popular variety with broad market prospects (Fukai and Udomdee 2005; Lu 2007; Mao et al. 2018; Taheri et al. 2012, 2014). At present, studies on the traits of *C. alismatifolia* have mainly focused on bract color (Koshioka et al. 2015), vase life (Kjonboon and Kanlayanarat 2005), inflorescence and flower initiation and development (Fukai and Udomdee 2005). As the most important ornamental trait of *C. alismatifolia*, bract color (Fig. 1) has begun to attract increasing attention among breeders and researchers. However, the mechanism underlying bract color formation remains unknown.

Although there are very limited studies on anthocyanins in bracts, anthocyanins on plant color have been well studied, given the importance of certain pigments in plant metabolism. The compounds involved in plant coloration (in addition to chlorophyll) are flavonoids (including anthocyanins), carotenoids, and betaine (Grotewold 2006). Anthocyanins are important secondary metabolites of plants and have important biological functions such as antioxidant and antibacterial effects and attracting insects for pollination (Winkel-Shirley 2001). In addition to their antioxidant and antibacterial activities, anthocyanins have been used as food colorants and pharmaceutical feedstock (Khoo et al. 2017; Xu et al. 2017). The biosynthesis of anthocyanins is catalyzed by several structural genes, such as *chalcone synthase* (*CHS*), and *flavonoid-3′,5′-hydroxylase* (*F3′5′H*), *dihydroflavonol 4-reductase* (*DFR*), and *anthocyanidin synthase* (*ANS*) (Belwal et al. 2020; Harborne and Williams 2000; Mol et al. 1998) and is also affected by transcription factors, such as MYB, bHLH, and WD40. Several subgroups of these transcription factors have been shown to be involved in anthocyanin synthesis, including subgroup 4–7, 44, and 79 of MYB regulators (Wu et al. 2022), subgroup IIIf, IIId + e, and IVd of bHLH regulators (Xie et al. 2012; Zhao et al. 2018, 2019, 2020), and WD40 proteins TRANSPARENT TESTA GLABRA1 (TTG1) homologs (Baudry et al. 2004; Belwal et al. 2020; Gonzalez et al. 2008). In the anthocyanin synthesis pathway of *Arabidopsis*, the *MYB* genes interact with the *bHLH* transcription factors and the *WD40* protein family to form the MYB–bHLH–WD40 (MBW) complex to regulate the formation and accumulation of anthocyanins (Dubos et al. 2010; Yan et al. 2021). In a previous qualitative and quantitative analysis of pigments in the pink bracts of *C. alismatifolia*, anthocyanidin malvidin 3-rutinoside was identified as the main pigment (Nakayama et al. 2000). The *CHS* and *DFR* genes have been cloned from *C. alismatifolia*, and the magenta color of the petals in *DFR* transgenic plants was more brilliant than that of the petals from wild type (Chanapan et al. 2017; Petchang et al. 2017). Moreover, *F3′5′H* was also identified as a key gene controlling anthocyanin synthesis in *C. alismatifolia* “Dutch Red” by transcriptome analysis (Li et al. 2022). In addition, most of the inner whorl bracts of *C. alismatifolia* have green tips with chlorophyll deposition and red pigmentation under them, which gives a desiccated appearance, thus reducing the ornamental value (Ding et al. 2021). Chlorophyll is a pigment that provides plants their characteristic green color and is mainly composed of chlorophylls *a* and *b*. Chlorophyll metabolism can be divided into three main steps, i.e., chlorophyll synthesis, chlorophyll cycle, and chlorophyll degradation, with each step being mediated by a series of important enzymes such as glutamyl-tRNA reductase (*HemA*), magnesium chelatase subunit H (*chlH*), and chlorophyllide a oxygenase (*CAO*) (Wang et al. 2020). Therefore, to clarify the formation of each color in *C. alismatifolia* bracts, the biosynthetic pathways of different pigments must be elucidated. Such knowledge will improve cultivar development through gene editing of gene targets in the pigment pathways.

At present, the traditional method for developing new varieties of *C. alismatifolia* is through hybrid breeding, which is costly and time intensive (Ke et al. 2020). Moreover, previous research on *C. alismatifolia* has mainly focused on the development of molecular markers without the availability of a complete genome reported for this species. A high-quality *C. alismatifolia* genome assembly will accelerate the development of new cultivars with desired traits. It will also improve the characterization of wild germplasms and aid in the discovery of novel genotypes and the identification of diversity hotspots for species conversation. Thus, it is urgent to compile a high-quality genome for *C. alismatifolia* to accelerate evolutionary studies as well as precision breeding and genome editing in the genus *Curcuma*.

Currently, only three genomes from Zingiberaceae have been published, including *Alpinia nigra* (Ranavat et al. 2021), *Z. officinale* (Cheng et al. 2021b; Li et al. 2021), and *C. longa* (Chakraborty et al. 2021), of which *C. longa* is only a draft genome. Here, we present the chromosome-scale assembly of *C. alismatifolia*, the first genome in *Curcuma*. The genome of *C. alismatifolia* was determined using a combination of high-accuracy long-read PacBio HiFi and proximity ligation Hi-C data. In total, a genome of 994.07 Mb was assembled with 95.25% of contigs anchored to 16 chromosomes, with an N50 of 57.51 Mb. We examined the patterns of whole-genome duplications as well as other duplication types and found that tandem and other small-scale gene duplications were important in the divergence of *C. alismatifolia* color morphs. In addition, we identified key genes involved in the anthocyanin and chlorophyll metabolism pathways in *C. alismatifolia* bracts that underlie this coloration. The publication of this reference genome and the genetic mechanisms controlling the color of *C. alismatifolia* bracts provide a valuable resource for the development of novel cultivars as well as increasing our understanding of the evolution of inflorescences among the monocots and providing an important genomic resource for clarifying the complex phylogenetic relationships in Zingiberaceae.

## Results

### *C. alismatifolia* genome assembly and annotation

The genome size of *C. alismatifolia* “Chiang Mai Pink” was estimated to be 1.10 Gb and the heterozygosity was found to be 1.7% using 87.45 Gb of MGI-SEQ 2000 survey data (Supplementary Figs. 1, 3, 4 and Table 1). This is slightly higher than the reported genome size of 998.5 Mb estimated by flow cytometry in a previous report (Mao et al. 2020). Then, 30.35 Gb of PacBio circular consensus sequence (CCS) reads were used for assembly, and 95.25% of the sequences were anchored to the 16 chromosomes by combining 110.73 Gb of Hi-C data, which was consistent with the expected number of chromosomes (2*n* = 32) (Leong-Skornickova et al. 2007), resulting in a genome of 994.07 Mb size (Fig. 1; Supplementary Fig. 2). The high fidelity of the genome assembly of *C. alismatifolia* was supported by the high mapping rates of 97.44% (MGI) and 99.07% (HiFi) (Supplementary Table 2). The high level of completeness of this assembly was also verified by a BUSCO (Simao et al. 2015) score of 96.53% (Supplementary Fig. 5) and a CEGMA (Parra et al. 2007) score of 95.16% (Supplementary Fig. 6). The long terminal repeat (LTR) assembly index (LAI) (Ou et al. 2018) score was 26.38 (Supplementary Fig. 7, Supplementary Table 3). These statistics suggest that the *C. alismatifolia* “Chiang Mai Pink” genome is a high-quality genome.

From the complete genome, 1,172,133 repeat units of different types were predicted, accounting for 75.84% (753,914,943 bp) of the total genome size (Supplementary Table 4). The long terminal repeats (LTR) accounted for the highest proportion of the genome (52.60%, Supplementary Table 4), among which the super families *Copia* (31.79%) and *Gypsy* (20.81%) dominated (Supplementary Table 4). A burst in LTR proliferation was inferred to have occurred 2.5 mya (Supplementary Fig. 8), with the genomic location of LTRs concentrated away from the SSR hotspots (Fig. 1). We identified 57,534 protein-coding genes (Supplementary Table 5) with a BUSCO score of 90.7% (Supplementary Table 6) and the gene numbers and repeat sequences in line with *C. longa* (Chakraborty et al. 2021) and the published transcriptome of *C. alismatifolia* (Taheri et al. 2019) (Supplementary Table 5). In addition, the total length of all genes (exons + introns) showed a similar pattern to that of other published monocot genomes (Supplementary Figs. 9, 10, 11 and 12). Up to 92.35% of the protein-coding genes have been annotated with KEGG, GO, NR, and other databases (Supplementary Table 7). In total, 9417 rRNAs, 8641 snRNAs, 1151 tRNAs, and 202 miRNAs were also annotated (Fig. 1, Supplementary Table 8). The GC content of the entire genome was approximately 43% (Fig. 1). There was a high GC content (~ 55%) region in chromosomes 13 (LG13) and 16 (LG16). These GC-enriched regions are the locations of the 45S ribosomal RNA, which are consistent with the high GC content of the internal transcribed spacers (ITS) for these genes in banana (Hribova et al. 2011). We also found that 5S rRNA genes were enriched in chromosome 1 (LG01), while tRNA-genes were enriched in chromosome 8 (LG08) (Fig. 1).

### Comparative genomics and whole-genome duplication (WGD) event

**Fig. 2:**
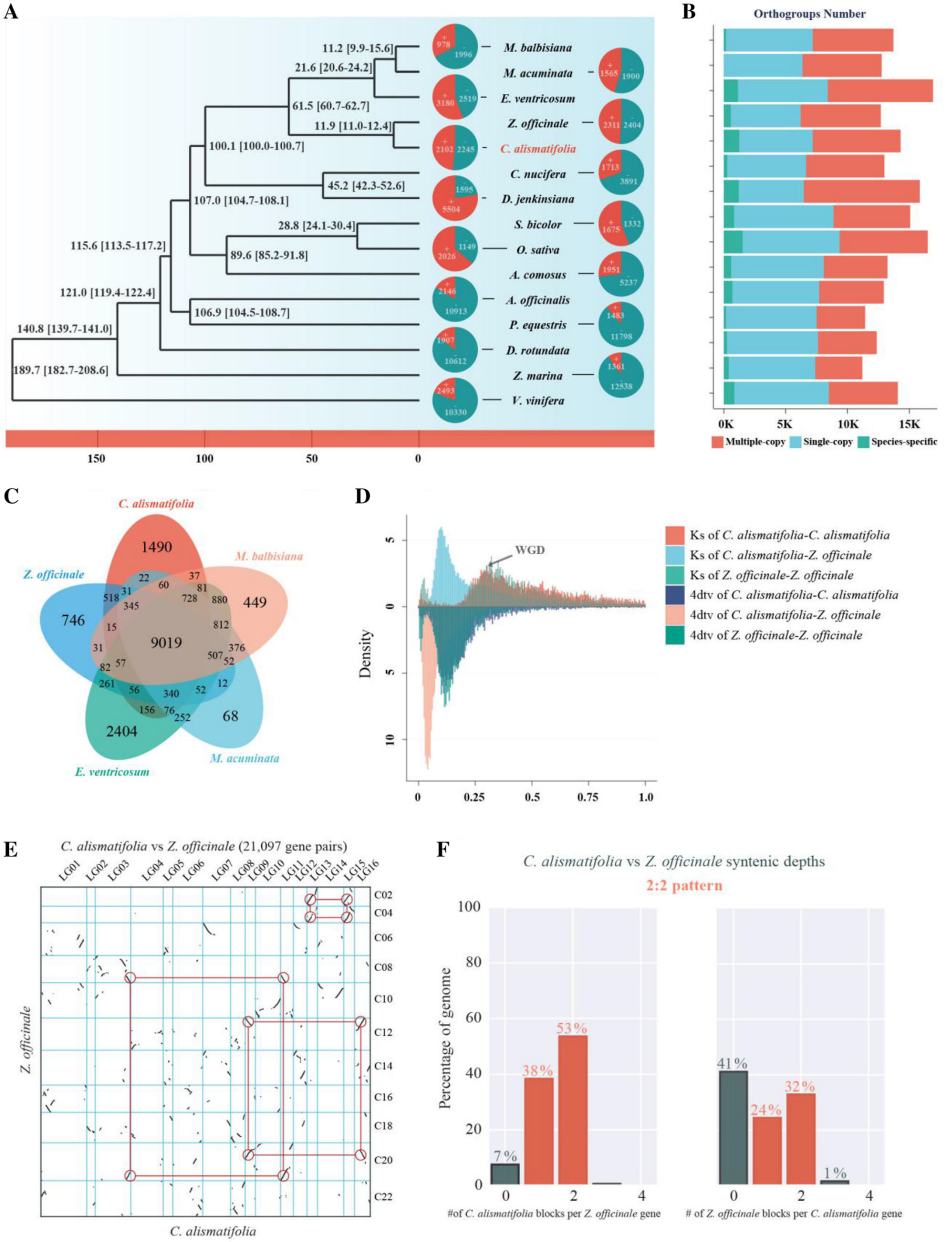
Evolution of the *C. alismatifolia* genome and gene families.** A** Phylogenetic tree constructed using maximum likelihood based on the concatenation of single-copy nuclear genes. **B** The distribution of orthogroups in each species. **C** Venn diagram of shared and unique gene families in Zingiberales species. **D** The distribution frequencies of synonymous substitutions (*Ks*) and substitutions of 4dtv sites. **E** Synteny patterns between *C. alismatifolia* and *Z. officinale*. **F** The 2:2 syntenic depth pattern between *C. alismatifolia* and *Z. officinale*

To clarify the phylogenetic position of *C. alismatifolia* and provide a general framework for understanding its genomic structure, genes of 217 single-copy gene families from 15 species, including *C. alismatifolia*, 13 other monocotyledonous species, and the outgroup grape (*Vitis vinifera*) were used for phylogenetic analysis. *C. alismatifolia* was inferred to have diverged from ginger (*Z. officinale*) around ~ 11.9 mya (Fig. 2A). A total of 9,019 gene families were shared by species in the Zingiberales (Fig. 2B, C, Supplementary Tables 9 and 10), among which *C. alismatifolia* had 1490 species-specific gene families (Fig. 2A). In the genome of *C. alismatifolia*, 2,102 gene families were found to be expanding, of which 120 expanded significantly (Fig. 2A). KEGG enrichment results showed that these expanded gene families were mainly related to environmental adaptation, such as plant–pathogen interaction, steroid hormone biosynthesis, and terpenoid backbone biosynthesis (Supplementary Figs. 13 and 14).

In the speciation and divergence of angiosperm lineages, WGD events are an important source of molecular diversity (Wu et al. 2020). The distributions of *Ks* and substitution rate of fourfold synonymous (degenerative) third-codon transversion (4dtv) sites of gene pairs in the collinear blocks of *C. alismatifolia* and *Z. officinale* indicated that both species have a shared *Ks* peak (Fig. 2D). This result was consistent with a previous report that *Z. officinale* has a shared WGD in Zingiberaceae (Cheng et al. 2021b). This WGD event was also supported by the collinear relationships between *C. alismatifolia* and *Z. officinale*, which had a 2:2 syntenic depth pattern shown by JCVI (Fig. 2E, F) and was further verified by the 1:2 pattern of collinearity between *C. alismatifolia* and *Amborella* (Supplementary Fig. 15). This result indicates that, compared with *Amborella*, a WGD event occurred in both *C. alismatifolia* and *Z. officinale* (Fig. 2E, F). We conducted the above analyses and reported validated evidence of an obvious WGD event in the *C. alismatifolia* genome*.*

### Gene duplication events contribute to the diversity of *C. alismatifolia*

**Fig. 3:**
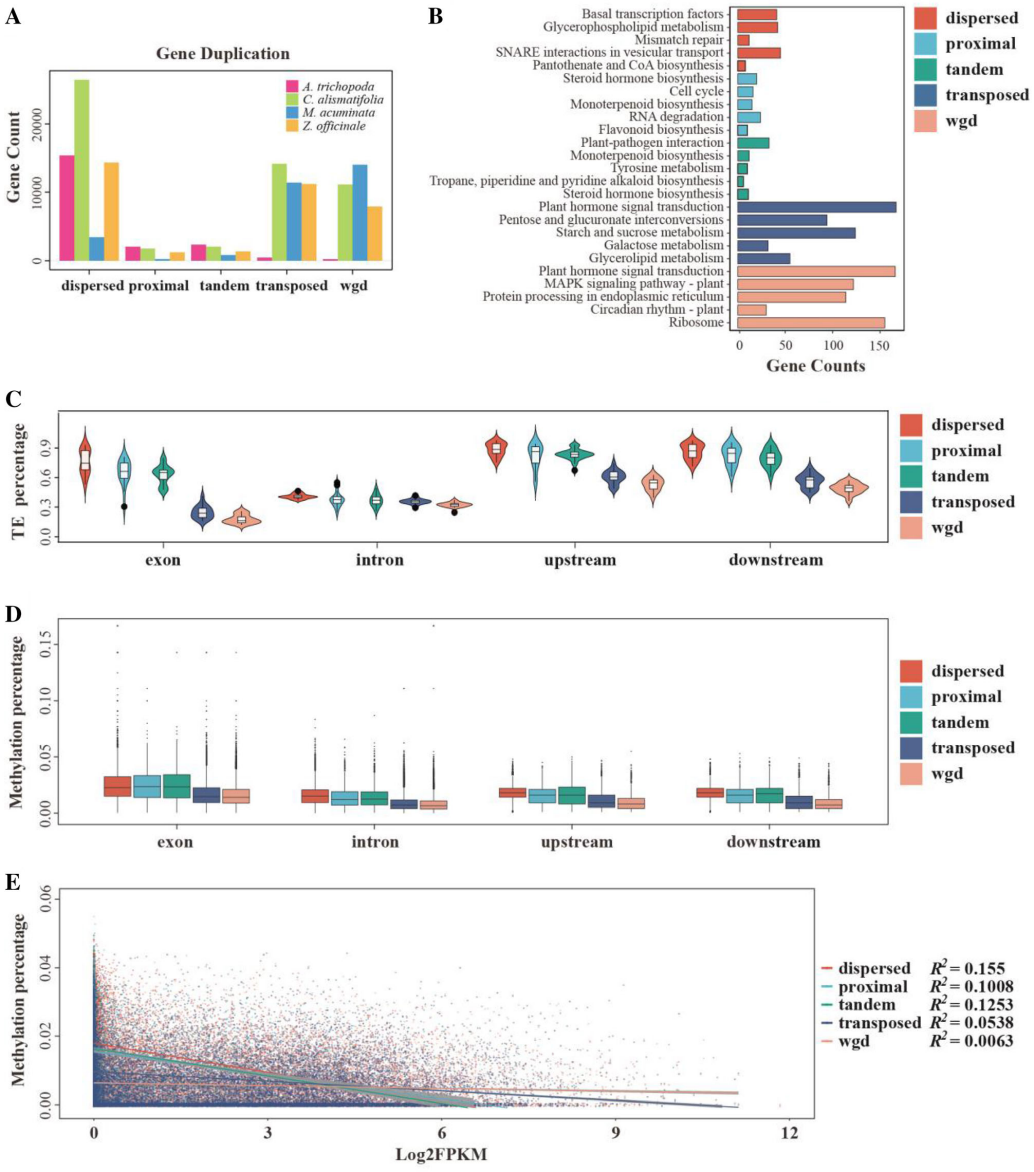
Gene duplication and evolution.** A** Genes derived from different modes of duplication in four different species. The gene types are whole-genome duplication (WGD), tandem duplication (TD), proximal duplication (PD), transposed duplication (TRD), and dispersed duplication (DSD). **B** Top five enriched pathways for each duplicated type in *C. alismatifolia* based on the KEGG analysis. **C** Percentage of different duplicated gene types which contain TEs in an exon, intron, and 1 kb upstream or downstream sequence from each CDS. **D** Percentage of cytosine methylation in different duplicated gene types in an exon, intron, and 1 kb upstream or downstream sequence from each CDS. **E** The relationship between methylation and gene expression among different types of duplicated genes

To investigate whether other gene duplication events played an important role in the evolution of diverse bract pigmentation, we identified five types of duplicated genes: 26,466 dispersed duplicates (DSD), 1777 proximal duplicates (PD), 2052 tandem duplicates (TD), 14,159 transposed duplicates (TRD), and 11,120 from whole-genome duplicates (WGD) (Fig. 3A, Supplementary Tables 11 and 12). We compared the *Ks* and *Ka*/*Ks* distributions from different types of duplicated genes and found that the DSD, PD, and TD gene pairs had higher *Ka*/*Ks* ratios and smaller *Ks* values than those of the other two types of duplicated genes (Supplementary Fig. 16). This result was similar to that of *Z. officinale*, but slightly different from the results of *M. acuminata* and the dicotyledonous *Rhododendron* (Yang et al. 2020) (Supplementary Fig. 16), wherein only PD and TD showed this pattern. Thus, these three types of duplicated genes in *C. alismatifolia* and *Z. officinale* had more rapid sequence divergence with stronger positive selection than the WGD and TRD genes. KEGG enrichment of the five types of duplicated genes also showed that these genes seem to be divided into two main categories, among which DSD, PD, and TD genes were enriched in one group for monoterpenoid biosynthesis and flavonoid biosynthesis, which might be related to species-specific differentiation. In comparison, the WGD and TRD genes in another group were associated with more conserved functions such as circadian rhythm and plant hormone signal transduction (Fig. 3B and Supplementary Fig. 17).

In addition, the function of replicated genes could be influenced by epigenetic processes such as DNA methylation, which has been shown to influence gene expression in certain taxa (Dyson and Goodisman 2020). We examined the distribution of the five duplicated gene types on chromosomes and found that genes of the TRD and WGD types were more concentrated at chromosome ends with an opposite positional trend to LTR distributions, whereas DSD, PD, and TD genes, especially DSD genes, showed a higher overlapping distribution with LTRs (Supplementary Fig. 18). The 5mC methylation of CG contexts detected by Nanopolish software showed that the distribution of methylation coincided with the distribution of LTRs and DSDs (Supplementary Fig. 18). Considering that TEs are known to promote gene replication (Bayer et al. 2020) and angiosperm chromosome remodeling (Douglas and Futuyma 2017), and that both TEs and methylation can affect gene expression, such as cytosine DNA methylation, which regulates TE silencing, imprinting, and gene expression (Bourque et al. 2018; Liu et al. 2018; Wang et al. 2019), we assessed their distribution in the genome. We hypothesized that TE and exon methylation might affect the distribution and expression of different classes of repetitive genes and contribute to their different evolutionary fates. Given this, we analyzed TE insertions and methylation in exons, introns, and 1 kb regions [the length of intergenic regions was longer than 1 kb for most genes (Supplementary Fig. 19)] of genes upstream and downstream in the five types of duplicated genes. As expected, more DSD, PD, and TD genes had TE insertions and higher methylation levels than the WGD and TRD genes (Fig. 3C, D, Supplementary Tables 13 and 14), especially in their exons and upstream and downstream regions (Fig. 3C and Supplementary Fig. 20). In addition, genes with TEs in their exons had lower relative expression levels (Supplementary Fig. 21). Moreover, TEs in the exons and the upstream and downstream regions had a higher degree of methylation than that in the other gene portions (Supplementary Fig. 22), which is consistent with a previous study (Zhang et al. 2018). However, to date, there have been no general conclusions regarding the effect of methylation in exon/intron/upstream/downstream regions on gene expression (Zhang et al. 2018). We found that the methylation levels in these four regions were approximately equivalent (Fig. 3D). Compared to the WGD and TRD genes, the DSD, PD, and TD genes had lower expression in multiple tissues and at different developmental stages (Supplementary Fig. 23). Therefore, the results showed that the higher the degree of methylation, the lower is the gene expression. The expressions of DSD, PD, and TD genes were more affected by methylation than WGD and TRD (Fig. 3E). DSD, PD, and TD genes also had longer intron lengths and relatively higher TE content than the WGD and TRD genes (Fig. 3C and Supplementary Fig. 24).

### Bract pigmentation genes in *C. alismatifolia*

**Fig. 4:**
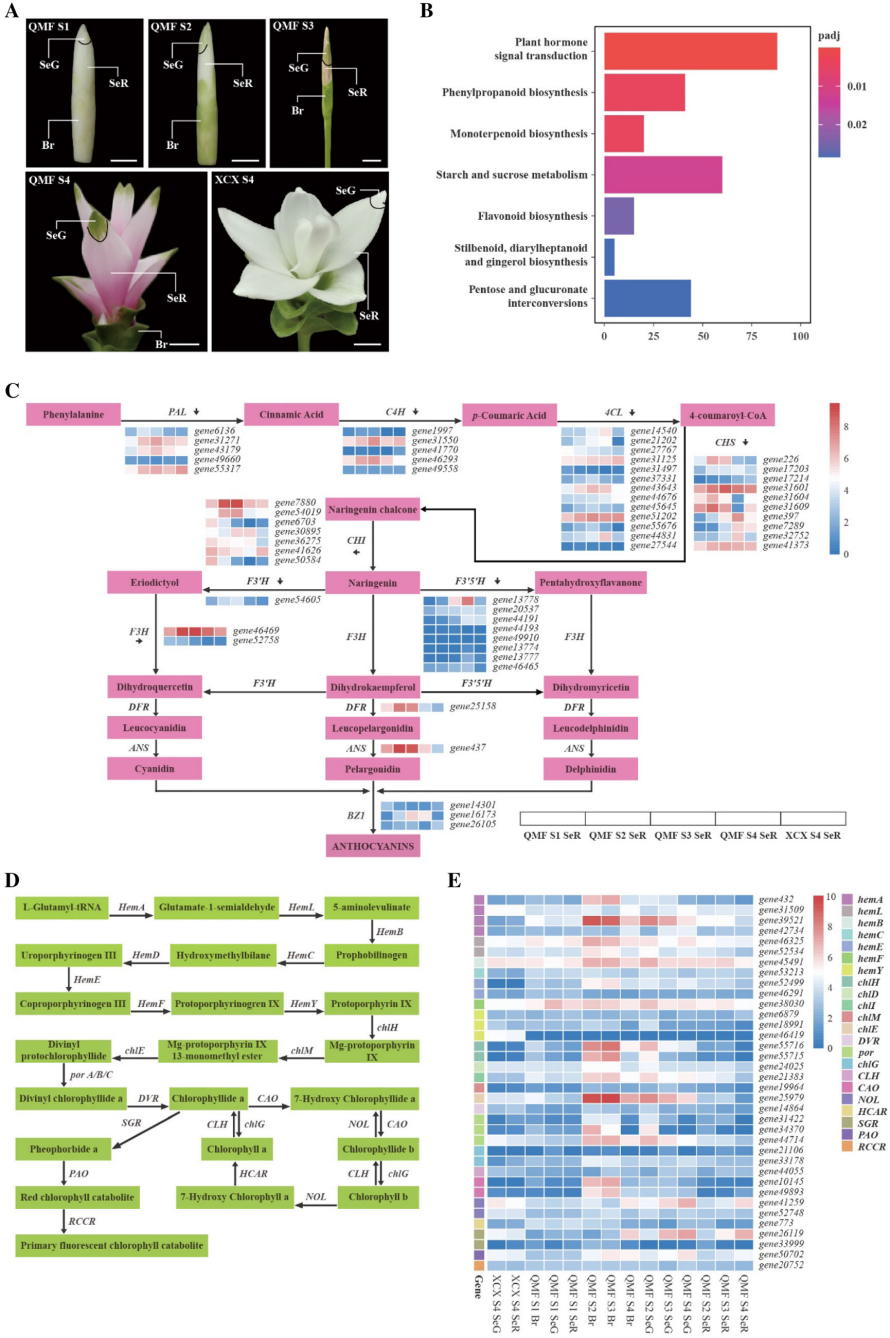
The anthocyanin and chlorophyll biosynthetic pathway genes identified by RNA-seq in the all-white bract morph *C. alismatifolia* and *C. alismatifolia* “Chiang Mai Pink”.** A** Locations of tissue samples for RNA-seq. Br (outer all-green bract), SeG (inner whorl bract tips), and SeR (inner whorl bract base) of *C. alismatifolia* “Chiang Mai Pink” (QMF) at different developmental stages and a white morph *C. alismatifolia* “Country Snow” (XCX) at S4 (bar of S1: 0.2 cm, bar of S2: 0.3 cm, bar of S3: 0.4 cm, bar of S4: 1.5 cm). **B** KEGG enrichment of DEGs in the QMF SeR vs XCX SeR at S4 stage. **C** Anthocyanin biosynthetic pathway in *C. alismatifolia*. Heatmaps show the FPKM with Log2 transformation of genes in SeR of QMF at S1–S4 stages and XCX at the S4 stage. Enzyme abbreviations: *PAL* phenylalanine ammonium lyase, *C4H* cinnamate-4-hydroxylase, *4CL* 4-coumaroyl-CoA synthase, *CHS* chalcone synthase, *CHI* chalcone isomerase, *F3H* flavanone 3-hydroxylase, *F3′H* flavonoid-3′-hydroxylase, *F3′5′H* flavonoid-3′,5′-hydroxylase, *DFR* dihydroflavonol 4-reductase, *ANS* anthocyanidin synthase, *BZ1* anthocyanidin 3-*O*-glucosyltransferase. **D** Chlorophyll biosynthetic and degradation pathway in *C. alismatifolia*. Enzyme abbreviations: *HemA* glutamyl-tRNA reductase, *HemL* glutamate-1-semialdehyde 2,1-aminomutase, *HemB* porphobilinogen synthase, *HemC* hydroxymethylbilane synthase, *HemD* uroporphyrinogen-III synthase, *HemE* uroporphyrinogen decarboxylase, *HemF* coproporphyrinogen III oxidase, *HemY* protoporphyrinogen/coproporphyrinogen III oxidase, *chlH* magnesium chelatase subunit H, *chlM*, magnesium-protoporphyrin O-methyltransferase, *chlE* magnesium-protoporphyrin IX monomethyl ester, *por* protochlorophyllide reductase, *DVR* divinyl chlorophyllide a 8-vinyl-reductase, *CAO* chlorophyllide *a* oxygenase, *chlG* chlorophyll/bacteriochlorophyll a synthase, *NOL* chlorophyll(ide) *b* reductase, *HCAR*, 7-hydroxymethyl chlorophyll a reductase, *CLH* chlorophyllase, *SGR* magnesium dechelatase *PAO* pheophorbide a oxygenase, *RCCR* red chlorophyll catabolite reductase. **E** Expression heatmaps of genes in the chlorophyll biosynthetic and degradation pathway

To investigate the mechanisms underlying the important trait of bract color in *C. alismatifolia*, a stable all-white bract morph, two cultivars of *C. alismatifolia*, i.e., “Country Snow” (XCX) and “Chiang Mai Pink” (QMF) were selected to conduct comparative transcriptomic analyses. Light microscopic observation showed that the color of the inner whorl bract tips of QMF was a mix of red and green colors (Supplementary Fig. 25), and the anthocyanin content of its inner whorl bract bases at S4 stage was 0.2338 mg/g fresh weight (FW). Tissue samples were collected from the outer all-green bract (Br), tip (SeG), and base (SeR) of the inner whorl ornamental bract at four developmental stages, i.e., including the early stage of bract initiation (S1), tip coloring stage (S2), base color transition stage (S3), and color change completion stage (S4), and used for transcriptomic analyses (Fig. 4A).

We identified the differentially expressed genes (DEGs) of SeR at S4 in the QMF vs XCX, which contained a total of 3347 upregulated and 3350 downregulated genes in QMF (Fig. 4B, Supplementary Table 15). KEGG enrichment analysis showed that these DEGs were enriched in the phenylpropanoid and flavonoid biosynthesis pathways (Fig. 4C), both of which are upstream of anthocyanin synthesis.

Based on previous reports (Belwal et al. 2020; Ferreyra et al. 2012) and our genome annotations, we analyzed the expression levels of all genes in the anthocyanin synthesis pathway in different samples (Belwal et al. 2020; Ferreyra et al. 2012) (Supplementary Fig. 26, Supplementary Table 16). We found that the tandem duplicated (TD) gene *F3′5'H* (*gene13778*) in QMF was highly expressed in SeR at the S3 and S4 stages, but lowly expressed at the S1 and S2 stages. This gene was minimally expressed in the different tissues of XCX (Fig. 4D, Supplementary Table 11). In addition, the genes *DFR* (*gene25158*), *ANS* (*gene437*), and *BZ1* (*gene16173*) were also significantly downregulated in XCX (Fig. 4D), among which *DFR* and *ANS* had a higher expression level and their expression patterns were similar to the bract color accumulation in the pink bracts. In addition, transcription factors were identified using iTAK (Supplementary Fig. 27 and Supplementary Tables 17 and 18). The subgroups of 4–7, 44, and 79 of MYB regulators (Wu et al. 2022), IIIf, IIId + e, and IVd of bHLH regulators (Xie et al. 2012; Zhao et al. 2018, 2019, 2020) and WD40 protein TTG1 homologs (Baudry et al. 2004; Belwal et al. 2020; Gonzalez et al. 2008) have been reported to be involved in anthocyanin synthesis; these most important subgroups of *MYB*, *bHLH*, and *WD40* genes (Supplementary Figs. 28 and 29, Supplementary Table 19) were identified by a phylogenetic analysis based on homologs from *A. thaliana* and SG79 in other species (Wu et al. 2022). On this basis, a total of 12 candidate regulators were screened out (Supplementary Fig. 30A, Supplementary Table 20) and further narrowed down to eight genes according to the weighted correlation network analysis (WGCNA) (Supplementary Fig. 30B and 31). Finally, ten genes, including *DFR*, *ANS*, 2 *MYB* (SG7 subgroup of *MYB*: *gene39947*, SG44 subgroup of *MYB*: *gene20923*), and 6 *bHLH* (IVd subgroup of *bHLH*: *gene46097*, IIId + e subgroup of *bHLH*: *gene29974*, *gene45529*, *gene40971*, *gene51401*, and IIIf subgroup of *bHLH*: *gene32335*) genes were verified by qRT-PCR. The qRT-PCR results showed that the expression patterns of these genes were consistent with the bract coloring period in the RNA-seq results (Supplementary Fig. 30c). Therefore, we believe that these genes play a crucial role in anthocyanin synthesis and are also closely related to the formation of white bracts, because the qRT-PCR results showed that these genes were not expressed in the SeR of white bracts.

Moreover, population structure analysis based on 56 *C. alismatifolia* cultivars showed that these samples were mainly divided into two groups, among which the inner bracts of samples in Group 2 possessed a similar morphology and color in the outer bracts, while there were differences between the inner and outer bracts of samples in Group 1 (Supplementary Fig. 32A). Principal component analysis (PCA) results also showed differentiation between these two groups (Supplementary Fig. 32B). To further verify the differences in bract color at the population level, one population with red inner whorl bract bases (SeR) (13 individuals) and one with white or green SeR(14 individuals) were selected to calculate *Fst*, and the results of the red and non-red populations further confirmed that 8 of the 14 candidate genes (*gene25158*, *gene437*, *gene318*, *gene39947*, *gene29974*, *gene45529*, *gene32394*, and *gene32335*) were differentiated at the population level (Supplementary Fig. 32C).

A previous study reported that the inner whorl bract tips (SeG) are green in *C. alismatifolia*, which is a phenomenon of chlorophyll deposition (Ding et al. 2021) (Fig. 4A). The results of WGCNA, a widely used transcriptome analysis method (Langfelder and Horvath 2008), revealed one *chlH* (*gene55716*) and one *CAO* (*gene49893*) gene in chlorophyll synthesis as the hub genes in the module most associated with the green trait (Supplementary Figs. 33 and 34, Supplementary Table 22). To understand the molecular mechanism of chlorophyll synthesis, we analyzed the expression patterns of all chlorophyll synthesis and metabolic pathway genes in different tissues (Fu et al. 2021; Wang et al. 2020) (Fig. 4E, Supplementary Table 21). We found that the downstream genes of the TD gene *chlH* (*gene55716*, *gene55715*) were minimally expressed in the white samples (Fig. 4E), suggesting that the *chlH* is a key gene controlling chlorophyll synthesis in *C. alismatifolia* bracts.

In addition to bract color, floral scent is an important economic trait of ornamental plants. Previous observation showed that different cultivars had different floral scents, and the “true” flowers of *C. alismatifolia* were the main sources of floral scents rather than its colorful bracts. Therefore, GC–MS was used to analyze the floral scents originating from the flowers of *C. alismatifolia* “Chiang Mai Pink” and revealed its volatile compounds, sesquiterpenoids and monoterpenoids, associated with floral scent, since the duplicated genes were mainly enriched in monoterpenoid biosynthesis (Supplementary Fig. 35A). Further analysis of the transcriptomes at different flower developmental stages revealed that *TPS*-a and *TPS*-b genes, which are mainly related to sesquiterpenoid and monoterpenoid synthesis, were downregulated as the flowers developed, implying that this kind of aroma will fade with full flowering; however, more evidence is needed for other aromatic substances (Supplementary Fig. 35B–C).

### Bulked segregant analysis (BSA) isolates the key genes related to bract color of *C. alismatifolia*

**Fig. 5:**
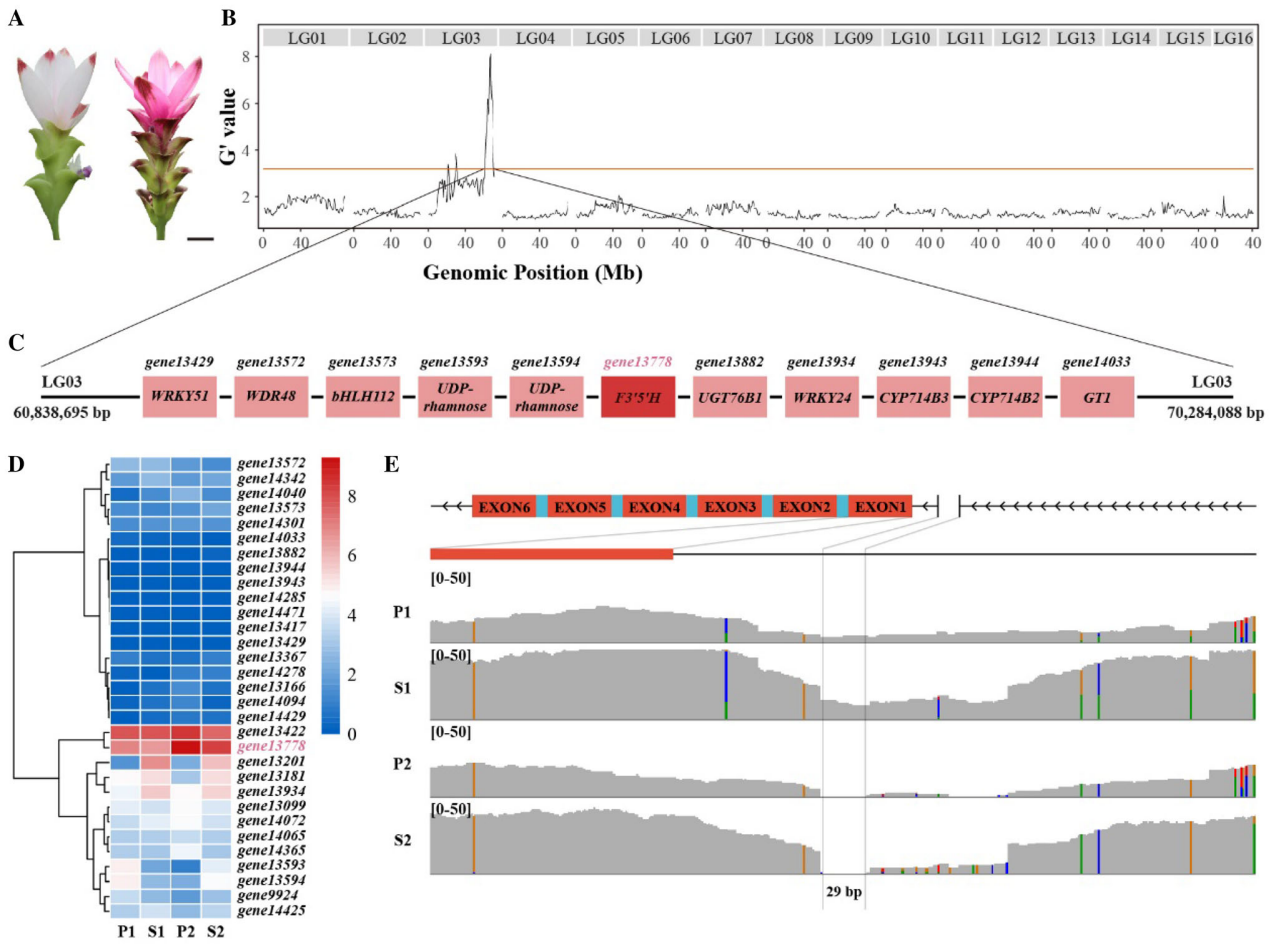
BSA-seq reveals the *F3′5'H* gene as a candidate gene responsible for red bract pigmentation of *C. alismatifolia*.** A** Left: *C. alismatifolia* “LM”; right: *C. alismatifolia* “JL”. Bar: 3 cm. **B** Genome-wide *G*´ value for allele frequency of SNPs between F1 hybrids S1 (LM) and S2 (JL) pools. **C** Anthocyanin biosynthesis related genes in BSA signal regions. **D** Expression heatmap of 31 anthocyanin biosynthesis-related genes from BSA signal regions. **E** Gene structure of candidate gene according to genome annotation of QMF and sequence depth in parents LM (P1) and JL (P2), and F1 hybrids LM (S1) and JL (S2)

To further understand the molecular mechanism of bract pigmentation, we sequenced two bulked populations with different bract colors (pink and red), each consisting of 50 offspring from an F1 population containing 985 individuals from a cross between the red line (*C. alismatifolia* “Scarlet” (JL), female parent with red bract) and pink line (*C. alismatifolia* “Dawn” (LM), male parent with pink bract), to a depth of 50 × (Fig. 5A). DNA and RNA from four samples [P1(LM), P2(JL), S1(LM), and S2(JL)] were sequenced for BSA and bulked segregant RNA-seq (BSR) analysis.

We identified SNPs between the two parental lines and computed the SNP index for the red line and pink line bulked populations as well as their differences (G' value) using a 1000 kb sliding window with a step size of 10 kb, as described by Mansfeld and Grumet (2018). Three genomic regions containing 1547 genes contributing to bract color, with a confidence threshold exceeding 95% were identified (Supplementary Table 23). Similar results were obtained in the BSR analysis (Supplementary Fig. 36 and Supplementary Table 24). Based on annotation information (Supplementary Table 25), we identified 31 genes that were potentially related to anthocyanin synthesis (Fig. 5B–D, Supplementary Table 26). Among them, three candidate genes, including one *F3′5′H* gene (*gene13778*) and two *MYB* genes (SG14: *gene13102* and SG4: *gene14458*) in QTL1, were identified for bract pigmentation based on gene expression, single nucleotide variants and structural variations (Fig. 5D, Supplementary Table 25). The promoter of *F3′5'H* gene had a 29 bp heterozygous region 94 bp upstream of the start codon in LM, and this same region had zero sequencing depth in JL (Fig. 5E). In addition, the second intron of *F3′5′H* gene showed an abnormal length with a heterozygous deletion of approximately 16.8 kb in length according to the genome annotation of QMF (Supplementary Fig. 37a). For the two *MYB* genes, the allele frequencies of the SNP in *gene13102* promoter at LG03_61315110 and LG03_61315135 in S1 of LM and S2 of JL were different. The SNP at LG03_61315110 in S1 of LM was C/A (55%/45%), whereas in S2 of JL pool was C (100%). The SNP at LG03_61315135 in S1 of LM was A/T (53%/47%), whereas in S2 of JL pool, it was A (100%) (Supplementary Fig. 38). The allele of the SNP in *gene14458*’ exon at LG03_69765180 was A/G (63%/38%), whereas in the S2 of JL pool, it was A (100%), implying that the structural variations in these genes may be responsible for the red or pink bract color (Supplementary Fig. 39). Furthermore, two *MYB* genes, four *bHLH* genes, seven *WRKY* genes, and two *WD40* genes were found to be differentially expressed in P2 vs. P1 and S2 vs. S1 based on the RNA data of P1(LM), P2(JL), S1(LM), and S2(JL) (Supplementary Tables 27 and 28). Among the four *bHLH* genes (*gene21747*, *gene41577*, *gene46097*, and *gene55364*), *gene55364* belongs to the IIId + e subgroup of *bHLH*, while *gene46097* belongs to the IVd subgroup of *bHLH* (Supplementary Table 19), which have been previously reported to be involved in the biosynthesis of anthocyanins.

## Discussion

### Monocotyledons have more complex WGD events

Here, we present a high-quality chromosome-scale genome assembly of *Curcuma* species and identify an obvious WGD event, as well as other duplicated genes, with an impact on the diversity of *C. alismatifolia* that promotes the coloration of bracts. Gene duplication plays an important role in evolutionary adaptation by providing “new” genetic material to the genome (Dyson and Goodisman 2020), and the WGD events are regarded as an important source of such diversity (Wu et al. 2020). It is now clear that the genomes of extant seed plants and angiosperms have undergone multiple WGD events and share an ancient polyploid ancestor. In addition, some angiosperms have undergone repeated independent whole-genome duplication events in recent times. For example, the banana (*Musa acuminata*) has three independent WGD events (D'Hont et al. 2012). Here, by identifying the collinear blocks of *C. alismatifolia* and *Z. officinale*, we found that *C. alismatifolia* and *Z. officinale* have a 2:2 collinear relationship (Fig. 2E, F), together with the *Ks* distribution, suggesting that one WGD event occurred in the ancestor of these two species (Fig. 2D–F). The WGD events in monocots are complex (Jiao et al. 2014; Wu et al. 2020). Previous studies have also indicated that ancestors of *Z. officinale* may have undergone multiple WGD events (Cheng et al. 2021b; Li et al. 2021). We further confirmed these WGD events with the fitted *Ks* results of WGDI for *C. alismatifolia* and the corresponding *Ks* distribution of *C. alismatifolia* with *Amborella* (Supplementary Figs. 40 and 41), combined with the weak signals in the median *Ks* distribution of collinear blocks of the *Z. officinale* paralogous gene pairs in WGDI collinear blocks (Supplementary Fig. 42). From this, it appears that both *C. alismatifolia* and *Z. officinale* have an additional WGD weak signal, which was further supported by the results of Tree2GD, suggesting WGD should have occurred once before the divergence of Zingiberaceae and Musaceae as well as once before the divergence of *C. alismatifolia* and *Z. officinale* (Supplementary Fig. 43 and 44). Sampling of more species in this group is needed to support the inferred additional WGD event. Based on the results of TreePL and MCMCtree, the most recent WGD event occurred after the divergence of *C. alismatifolia* and *Z. officinale* ancestors from ancestors of *Musa* and before the divergence of *C. alismatifolia* with *Z. officinale* (Fig. 2A and Supplementary Fig. 45).

### Gene duplication as a key to the evolution of diverse bract pigmentation

Five duplicated gene types were identified and compared in *C. alismatifolia*, *Z. officinale*, and *M. acuminata* (Fig. 3A). In previous studies of the dicotyledonous plant *Rhododendron*, TDs and PDs were found to contribute to an increase in the ratio of enzymatic genes in the anthocyanin biosynthesis pathway, suggesting that TDs and PDs are important in the evolution of flower color diversity (Yang et al. 2020). In our study, we found that the TD and PD genes of Zingiberales were significantly enriched in the upstream steps of the anthocyanin synthesis pathway. In terms of TE content, degree of methylation, *Ks*, and gene expression, TD, PD, and DSD were more similar to WGD or TRD genes (Fig. 3C–E, Supplementary Figs. 16, 17, 18, 19, 20, 21, 22, 23 and 24). The Venn diagram of these five types of genes shows that TD and PD share 117 genes, whereas WGD and TRD share 4639 genes, indicating that DupGen_finder (Qiao et al. 2019) may not be able to clearly discern duplicates of these types in all cases or that terms used to define these duplicate types can overlap. For instance, no such overlap was found among the other groups, indicating that some duplicates likely fulfilled the criteria for two categories, such as a WGD that has been transposed (Supplementary Fig. 46). Overall, TD, PD, and DSD can be regarded as a general class and appear to be involved in the evolution of color diversity in *C. alismatifolia* bracts. Here, we also found that there was no significant difference in the methylation levels between exons, introns, or upstream or downstream regions of different categories of duplicated genes (Fig. 3D). However, WGD and TRD genes had lower levels of methylation and higher gene expression (Fig. 3D and Supplementary Fig. 23) and were involved in growth and development processes (Fig. 3B and Supplementary Fig. 17), suggesting that genes retained after WGD or TRD duplication are more often related to conserved functions. This result is consistent with previous studies, where the retention and loss of repetitive genes after WGDs are not random, but have a bias for genes related to signal transduction, transcription factors, and genes related to development. The existence of gene retention bias may result from the functional divergence of genes related to adaptation to novel environments (Wu et al. 2020). However, how DNA methylation affects the evolution and retention of these two types of duplicated genes requires further study.

### A complex regulatory mechanism controls the color diversity in *C. alismatifolia* bracts

Here, we also identified that *F3′5'H*, *DFR*, and *ANS* are key genes in the anthocyanin biosynthesis pathway, and transcriptome analysis revealed that *DFR* and *ANS* play a more critical role, and the role of *DFR* gene in *C. alismatifolia* has also been verified (Petchang et al. 2017). Several regulatory factors were identified to be closely related to *DFR* and *ANS*, among which the SG7 subgroup of *MYB gene39947* (Supplementary Fig. 47) had a stronger correlation with *DFR* and *ANS*, and qRT-PCR results verified that it has a very similar expression trend as *DFR* and *ANS*. A *TRANSPARENT TESTA 8* (*TT8*) homologous gene, *gene32335*, was also identified (Supplementary Fig. 48). TT8 is considered sufficient for the expression of *DFR* and *ANS* genes and is reported to be one of the key regulators of anthocyanin production in many plant species (Yan et al. 2021). In addition, previous reports revealed that *LcbHLH92a* and *LcbHLH92b* in sheepgrass are involved in anthocyanin and proanthocyanidin synthesis (Zhao et al. 2019). In our study, an *AtBHLH92* homologous gene, *gene46097* (Supplementary Fig. 48), was simultaneously identified by BSA and transcriptome analysis in our study, suggesting a crucial role. Therefore, we believe that there is a complex mechanism controls bract color formation. WGCNA has predicted a regulatory network, which also provides a basis for subsequent experimental verification. In addition, candidate gene *F3′5'H* (*gene13778*), which is a flavonoid-3',5'-hydroxylase, is regarded as a member of the cytochrome P450 family and is a crucial enzyme required for producing blue or purple flowers was identified (Hopkins and Rausher 2011; Shimada et al. 1999). Our study also showed that the *F3′5'H* gene (*gene13778*) has a long length in *C. alismatifolia* “Chiang Mai Pink” (Supplementary Fig. 37A), mainly due to the second intron being over 20 kb (verified by mapping HiFi reads) as well as a 16.8 kb heterozygous deletion (Supplementary Fig. 37A). Previous studies have found that plant introns are generally shorter than those of animals, even in species with large genomes (Jin 2007), making the *F3′5'H* gene (*gene13778*) in *C. alismatifolia* an extreme outlier to this general pattern. We also found that the copy number of *F3′5′H* gene varied in Zingiberales; *C. alismatifolia* had 8 *F3′5'H* genes, *Z. officinale* had 6 *F3′5′H* genes, and *M. acuminata* had 4 *F3′5'H* genes according to gene annotations, among which the *F3′5′H* gene in *Z. officinale* had a length of 12,746 bp with 1040 amino acids (*gene13778* had 957 amino acids), as well as six exons, and its longest intron had a length of 8,702 bp (Supplementary Fig. 37B, C). The phylogenetic tree showed that *gene13778* also clustered with *F3′5′H* genes in *Petunia* (Supplementary Fig. 37B), which also confirmed the identity of this gene. However, considering the complexity of this region, where multiple *F3′5′H* genes repeat in tandem, and the limitations of existing gene structure prediction software, we believe that more experimental evidence is needed in the future to verify the structure of *F3′5′H* gene and reveal the differences in the expression levels of different transcripts, even the divergence within *C. alismatifolia* cultivars. In summary, the key genes involved in anthocyanin synthesis pathways were identified, and resequencing analyses found that genetic differentiation was associated with different bract color groups, concluding that color formation in *C. alismatifolia* bracts is a complex process, which also needs to be verified by further studies.

## Conclusions

Our study found that different categories of duplicated genes in *C. alismatifolia* genome were diversified in function by the duplicate type. This includes the evolution of different colored bracts of *C. alismatifolia* through the tandem duplication of genes and subsequent changes in gene structure. We identified the key anthocyanin synthesis genes *DFR, ANS* and *F3′5′H* and chlorophyll synthesis genes *chlH* and *CAO* in the formation of bract color and inferred the potential contribution of individual members of the transcription factor gene families. These results provide a basis for further identification of gene function in *C. alismatifolia* and related species. In conclusion, the reference genome of *C. alismatifolia* presented in this study provides a key resource for further studies and development of novel cultivars through marker-assisted breeding and genome editing.

## Materials and methods

### Plant materials

The *C. alismatifolia* cultivar “Chiang Mai Pink” was selected for whole-genome sequencing and assembly. The cultivar was planted in the greenhouse at Shenzhen Institute of Agricultural Genomics, Chinese Academy of Agricultural Sciences. Leaf tissue was used for whole-genome sequencing, while flowers, leaves, and young stems were subjected to RNA sequencing (RNA-seq) to support genome annotation and analyze of gene expression levels (Supplementary File 1).

### Library preparation and sequencing

We extracted the DNA from leaves by following the procedures of Qiagen Genomic DNA kit. According to the standard protocol of PacBio, 20 kb preparation solution was used to obtain the SMRTbell target size library (Pacific Biosciences, CA, USA), and then the HiFi data was generated with CCS software (https://github.com/PacificBiosciences/ccs). Genomic DNA (1–1.5 μg) was randomly interrupted into 200–400 bp fragments and sequenced on the MGI-SEQ 2000 platform. The total RNA of all sample materials were extracted with RNAprep pure Plant Kit (TIANGEN), and the RNA sequencing was carried out by MGI-SEQ 2000 sequencing platform. The Hi-C library was constructed with *Dpn*II restriction enzyme and sequenced on MGI-SEQ T7. The Fastp v0.19.4 was used to perform quality control (Chen et al. 2018).

### K-mer analysis and genome assembly

The Jellyfish v2.3.0 program (Marcais and Kingsford 2011) and Kmerfreq (Liu et al. 2013) were used to conduct the k-mer analysis by using the MGI data to estimate the genome size Genomes were assembled by using 30.35 Gb of high-quality HiFi reads using hifiasm v0.12 software with default parameters (Cheng et al. 2021a) (https://github.com/chhylp123/hifiasm), leading to a 1.22 Gb preliminary assembled genome. Using the quality-controlled MGI data, the genome was polished using Nextpolish v1.2.4 software (Hu et al. 2020) for four iterations, and the corrected genome was compared with the nr/nt database (NCBI) to remove possible sequences originating from biological contamination (such as endophytes).

### Hi-C scaffolding

A total of 109.71 Gb clean paired-end reads generated from Fastp v0.19.4 (Chen et al. 2018) were mapped to the 1.19 Gb preliminary assembled genome by using bowtie2 v2.3.2 (Langmead and Salzberg 2012), then the unique mapped reads were obtained. HiC-Pro v2.8.1 (Servant et al. 2015) identified and retained valid interactive paired reads from unique reads described above for further analysis. LACHESIS (Burton et al. 2013) (https://github.com/shendurelab/LACHESIS) was used to further aggregate, sequence, and locate scaffolds onto the chromosomes. Finally, the errors of placement and orientation were corrected with manual adjustment. The final chromosome anchor rate was 95.25%.

### Genomic evaluation

The integrity of the assembled genome was assessed by BUSCO v4.0.5 (Simao et al. 2015) based on single-copy homologous genes in the OrthoDB database embryophyta_odb10. CEGMA v2 (Parra et al. 2007) was also used to predict the genome completeness based on its database. HISAT2 v2.2.1 (Kim et al. 2019) was used to map the RNA-seq data from flowers, pedicels, and leaves, BWA v0.7.17-r1188 (Li and Durbin 2010) was used to map the MGI data, and minimap2 v2.21-r1071 (Pertea et al. 2016) was used to map the HiFi data to genome to calculate the mapping rate.

### Repetitive sequence annotation

The genome repeat sequence annotation was conducted by using the Extensive de novo TE Annotator (EDTA) v1.9.4 (Su et al. 2021), a toolkit for de novo annotating TEs in whole-genome datasets. The LAI and insertion time were calculated by using LTR_retriever v2.9.0 (Ou et al. 2018; Ou and Jiang 2018), with a substitution rate of default 1.3e-8 *Ks*/year.

### Gene structure and function annotation

Trinity v2.8.5 (Grabherr et al. 2011) was used to assemble the transcripts for predicting genes. Then the transcript-based predictions was conducted with PASA v2.4.1 (Haas et al. 2003). We also performed homology predictions by using the protein sequences of *M. balbisiana* (NCBI, GCA_004837865.1), *Z. mays* (Phytozome V13, v4), *O. sativa* (Phytozome V13, v7.0), and *Z. officinale* (NCBI, GCA_018446385.1), and mapped them to the genome of *C. alismatifolia*, with these homology annotation results being input to Augustus v3.3.3 (Stanke et al. 2008) for training. The genes from PASA v2.4.1 (Haas et al. 2003) were further used to train the GlimmerHMM v3.0.4 (Majoros et al. 2004), SNAP v2006-07–28 (Korf 2004) and Augustus v3.3.3 (Stanke et al. 2008) software to get results of de novo gene prediction. We used Evidencemodeler v1.1.1 (Haas et al. 2008) to integrate all above evidence, and the results were re-trained using PASA v2.4.1 (Haas et al. 2003) for one final round of gene annotation.

Blastp v2.9 (ftp://ftp.ncbi.nlm.nih.gov/blast/executables/blast+/LATEST/) was used to annotate the gene functions by comparing the protein sequences of *C. alismatifolia* with Swiss-Prot (uniprot_sprot), NR (https://ftp.ncbi.nlm.nih.gov/blast/db/FASTA/nr.gz) and KOG (https://ftp.ncbi.nih.gov/pub/COG/KOG/) databases with feature assignments made based on the best hits. Interpro annotation and GO annotation were performed by interproscan-5.21–60.0 (Blum et al. 2021). The KAAS server (https://www.genome.jp/kegg/kaas) was used to identify the KEGG pathway. For a more complete and comprehensive functional annotation, we employed egg-mapper v2.0.1 (Huerta-Cepas et al. 2017), to further perform the functional annotation based on eggNOG v5.0 (Huerta-Cepas et al. 2019). In addition, tRNAscan-SE v2.0.8 (Chan et al. 2021) was used to annotate tRNA, barrnap v0.9 (https://github.com/tseemann/barrnap) was used to annotate rRNA and cmscan v1.1.2 from the software suite Infernal (Nawrocki and Eddy 2013) was used to annotate other ncRNAs based on the Rfam database (Kalvari et al. 2021).

### Orthologous gene family identification, phylogenetic analysis, estimation of divergence time, and expansion and contraction of gene family expansion

By using OrthoFinder v2.5.2 (https://github.com/davidemms/OrthoFinder), we obtained orthogroups for 15 species (Supplementary Table 5), and the protein sequences of 217 single-copy orthogroups were obtained. The MAFFT v7.490 software with default settings (Katoh and Standley 2013) was used to perform sequence alignments for each single-copy gene family and the alignments were converted to a nucleotide matrix by pal2nal v14 (Suyama et al. 2006). Under the GTRGAMMA model, phylogenetic analysis was constructed with RAxML v8.2.12 (Stamatakis 2014). The MCMCTree program of the PAML v4.9 (Yang 2007) was further applied for estimation of species divergence time based on three soft bounds at three nodes (Cheng et al. 2021b). The expansion and contraction analyses was performed on the basis of the dated phylogeny tree and the homologous gene families from 15 species using the CAFE v4.21 program (Bie et al. 2006). GO and KEGG enrichment analyses were then performed with genes in significantly expanded families.

### Duplicated gene identification and WGD analysis

To study the size evolution of the *C. alismatifolia* genome, we identified whole-genome duplication events in *C. alismatifolia*. We identified five types of duplicated genes in *C. alismatifolia*, *Z officinale*, and *M. acuminata* by utilizing the DupGen_finder (Qiao et al. 2019) software with default parameters. WGDI (https://github.com/SunPengChuan/wgdi) was used to verify the WGD results. The program JCVI v1.1.18 (Tang et al. 2008) was used to further analyze the collinearity of *C. alismatifolia* and *Z. officinale*.

### Methylation analysis

Genomic DNA (2 μg) was obtained for ONT (Oxford Nanopore Technology) library preparations and then sequenced on the ONT PromethION sequencer. The call-methylation module of Nanopolish v0.13.2 (https://github.com/jts/nanopolish) was used to analyze 5-methylcytosine in the CG context in the genome based on Fast5 files, then the results were filtered according to the condition of methylated_frequency ≥ 0.5.

### Transcriptome analyses and gene co-expression networks

The clean RNA-seq reads from different sample were mapped to the *C. alismatifolia* genome with HISAT2 2.2.1 (Kim et al. 2019), and StringTie v2.1.6 (Pertea et al. 2016) was used to calculate the FPKM of genes in each sample with Log2 transformation, while using the Log2FPKM ≥ 0.5 cutoff for the gene in at least one sample to ensure that the gene was expressed. FeatureCounts v2.0.1 (Liao et al. 2014) was used to calculate the counts of each gene in each sample, then the differentially expressed genes (DEGs) of QMF SeR vs XCX SeR at the S4 period were analyzed by utilizing DESeq2 v1.34.0 software (Love et al. 2014). The following FC value range was used as the criterion for selecting DEGs: |log2FoldChange|≥ 1.5, adjusted *P* value ≤ 0.01. Based on the FPKM of genes, the R package WGCNA v1.70-3 (Langfelder and Horvath 2008) was used to build the co-expression network. Primers designed for qRT-PCR use were tested and listed in Supplementary Table 29. The Applied Biosystems™ PowerUp™ SYBR™ Green Master Mix (Thermo Fisher Scientific, US) was used for qRT-PCR with a CFX Connect™ Real-Time System (BIO-RAD, US). Two biological repeats and two technical repeats were carried out for each gene, and the relative expression level was calculated through the comparative 2^−ΔΔCT^ method.

### GO and KEGG enrichment

Based on the results from eggnog-mapper v2.0.1 (Huerta-Cepas et al. 2017, 2019) software, the protein sequences of *M. acuminata*, *Z. officinale* and *C. alismatifolia* were functionally annotated, and the GO and KEGG annotation results of the genes were extracted. With the help of the R package AnnotationForge v1.36.0 (https://bioconductor.org/packages/AnnotationForge/). The clusterProfiler v4.2.1 (Wu et al. 2021) program was used for GO and KEGG enrichment analysis. The visualization of enrichment results was generated with R package ggplot2 (https://github.com/tidyverse/ggplot2).

### Bulked segregant analysis

The *C. alismatifolia* “Scarlet” (JL*,* red line) and *C. alismatifolia* “Dawn” (LM, pink line) used for BSA were planted in the Environmental Horticulture Research Institute, Guangdong Academy of Agricultural Sciences. The individual plants of JL and LM grown under natural conditions were used for crossbreeding to obtain F1 hybrid populations. The segregation ratio was 502 (with the same red bract as JL): 483 (with the same pink bract as LM). Sequencing of the extracted DNA and RNA from parental LM (P1) and JL (P2), F1 hybrids LM (50 individuals mixed, S1) and JL (50 individuals mixed, S2) was carried on an Illumina NovaSeq 6000 platform. Data filtering and quality control were performed by Fastp v0.19.4 (Chen et al. 2018). BWA v0.7.17-r1188 (Li and Durbin 2010) and STAR v2.7.9a (Dobin et al. 2013) were used to map DNA and RNA data to the genome, separately, then GATK v4.2.2.0 (DePristo et al. 2011) was used for SNP calling. Finally, BSA and BSR analysis was performed using the R package QTLseqr v0.7.5.2 (Mansfeld and Grumet 2018). The quantitative method of transcript is the same as described in transcriptome analyses, and the R package edgeR v3.36.0 (Robinson et al. 2010) was used to analyze the differentially expressed genes of S2 vs S1 and P2 vs P1, using the following FC value range as the criteria for selecting DEGs: |logFC|≥ 1, FDR ≤ 0.01.

A detailed description of the above analysis, as well as the methods used for other analyses is listed in Supplementary File 1.

## Supplementary Information

Below is the link to the electronic supplementary material.

Supplementary file1 (DOCX 68 KB)

## Data Availability

The genome sequence data including HiFi, ONT, and Hi-C data were deposited in the Genome Warehouse in National Genomics Data Center (NGDC), under accession number CRA006523, while the whole-genome assembly was also deposited under accession number GWHBHOP00000000 that is publicly accessible at https://ngdc.cncb.ac.cn/gwh.
