# Supplementary material for: The genomic and bulked segregant analysis of Curcuma alismatifolia revealed its diverse bract pigmentation
Source: aBIOTECH. 2022 Oct 6;3(3):178–96. doi: 10.1007/s42994-022-00081-6 (PMC9590460; doi:10.1007/s42994-022-00081-6)
Supplement: Supplementary file 1 — Supplementary file1 (DOCX 68 KB) [file 42994_2022_81_MOESM1_ESM.docx]

**Supplementary File 1. The detailed materials and methods**

**Plant materials**

The most popular *C. alismatifolia* cultivar ‘Chiang Mai Pink’ was selected for whole genome sequencing and assembly. The cultivar was planted in the greenhouse at Shenzhen Institute of Agricultural Genomics, Chinese Academy of Agricultural Sciences. Leaf tissues were used for whole genome sequencing, while flowers, leaves, and young stems were subjected to RNA sequencing (RNA-seq) to support genome annotation and analyze of gene expression levels. Tissues were snap-frozen in liquid nitrogen and stored at -80°C for subsequent nucleic acid extraction. In addition, we also collected the tissue Br (outer all-green bract), SeG (inner whorl bract tips), and SeR (inner whorl bract base) of *C. alismatifolia* ‘Chiang Mai Pink’ (QMF) and *C. alismatifolia* ‘Country Snow’ (XCX) at different developmental stages (S1: 1cm, S2: 1.5cm, S3: 2cm, S4: 8cm), which containing QMF S1 Br (3 replicates), QMF S1 SeG (2 replicates), QMF S1 SeR (3 replicates), QMF S2 Br (3 replicates), QMF S2 SeG (3 replicates), QMF S2 SeR (3 replicates), QMF S3 Br (2 replicates), QMF S3 SeG (2 replicates), QMF S3 SeR (3 replicates), QMF S4 Br (3 replicates), QMF S4 SeG (2 replicates), QMF S4 SeR (2 replicates), XCX S4 SeG (3 replicates), XCX S4 SeR (3 replicates), XCX S4 SeG (3 replicates) and XCX S4 SeR (3 replicates). RNA sequencing (RNA-seq) was used to analyze differences in the mechanism of bract pigmentation.

**Library preparation and sequencing**

We extracted the DNA from leaves by following the procedures of Qiagen Genomic DNA kit. According to the standard protocol of PacBio, 20 Kb preparation solution was used to obtain the SMRTbell target size library (Pacific Biosciences, CA, USA). The CCS software (https://github.com/PacificBiosciences/ccs) was used to generate the HiFi data, and finally a total of 30.35 Gb CCS reads were obtained. The total number of reads was 2,107,601, the average read length was 14.40 kb, and the reads N50 was14.66 kb, of which the longest read length was 41.55 kb.

Genomic DNA (1-1.5μg) was randomly fragmented with Covaris, and the fragments with average size of 200-400bp were selected using AMPure XP-Medium kit for PCR purification, after which the library was prepared and sequenced on the MGI-SEQ 2000 platform. A total of 87.45 Gb raw data was obtained, that was then quality controlled and filtered by Fastp v0.19.4 (Chen et al. 2018), from which 72.67 Gb clean data was obtained.

The total RNA of all sample materials used for RNA sequencing were extracted with RNAprep pure Plant Kit (TIANGEN), and the RNA sequencing was carried out by MGI-SEQ 2000 sequencing platform, from which 6.21~9.23 Gb raw data was obtained.

To anchor the contigs to chromosomes, we extracted genomic DNA from leaves and treated it with *Dpn*II restriction enzyme. Then, the Hi-C library was constructed and 110.73 Gb of raw data was obtained using an MGISEQ-T7 platform. Fastp v0.19.4 was used to perform quality control (Chen et al. 2018) and 109.71 Gb of clean data was obtained.

**K-mer analysis and genome assembly**

To assess genome characteristics of *C. alismatifolia*, k-mer analysis was performed using the MGI data to estimate the genome size and heterozygosity. Briefly, quality-filtered reads were subjected to 17-mer frequency distribution analysis using the Jellyfish v2.3.0 program (Marcais and Kingsford 2011). By analyzing the 17-mer depth distribution from the 350-bp library with Kmerfreq (Liu et al. 2013), we evaluated genome size. In addition, we used short segment data from the *Arabidopsis* genome to simulate corresponding depths, and perform K-mer curve fitting under different gradient combinations of heterozygosity rates to estimate the heterozygosity rate of *C. alismatifolia*.

Genomes were assembled by using 30.35 Gb of high-quality HiFi reads using hifiasm v0.12 software with default parameters (Cheng et al. 2021a) (https://github.com/chhylp123/hifiasm), leading to a preliminary assembled genome version with a genome size of 1.22 Gbp. Using the quality-controlled MGI data, the genome was polished using Nextpolish v1.2.4 software (Hu et al. 2020) for four iterations, and the corrected genome was compared with the nr/nt database (NCBI) to remove possible sequences originating from biological contamination (such as endophytes). The corrected draft genome had a total length of 1.19 Gb and an N50 of 44.62 Mb.

**Hi-C scaffolding**

A total of 109.71 Gb clean data were used for Hi-C scaffolding. The low-quality sequences (quality scores < 20), adaptor sequences and sequences length < 30 bp were filtered out using Fastp v0.19.4 (Chen et al. 2018). Then, the clean paired-end reads were mapped to the 1.19 Gb assembled draft genome using bowtie2 v2.3.2 (Langmead and Salzberg 2012) (-end-to-end --very-sensitive -L 30) to get the unique mapped paired-end reads. HiC-Pro v2.8.1 (Servant et al. 2015) identified and retained valid interactive paired reads from unique reads described above for further analysis. Invalid read pairs, including dangling-end, self-cycle, re-ligation, and dumped products were filtered by HiC-Pro v2.8.1 (Servant et al. 2015). LACHESIS (Burton et al. 2013) was used to further aggregate, sequence, and locate scaffolds onto the chromosomes. After LACHESIS clustering, a total of 1,173.30 Mb of sequence was mapped to the 16 chromosomes, accounting for 98.95% of the total length, while the number of corresponding contig bins was 12,065, accounting for 98.89% of the draft genome. Finally, the errors of placement and orientation were corrected with manual adjustment. The final chromosome anchor rate was 95.25%.

**Genomic evaluation**

The integrity of the assembled genome was assessed by BUSCO v4.0.5 (Simao et al. 2015) (Benchmarking Universal Single-Copy Orthologs) based on single-copy homologous genes in the OrthoDB database embryophyta_odb10 (containing 1,614 BUSCO genes). CEGMA v2 (Parra et al. 2007) was also used to predict the genome completeness based on its database, and obtain the core gene information in the genome. HISAT2 2.2.1 (Kim et al. 2019) was used to map the RNA-seq data from flowers, pedicels, and leaves, BWA v0.7.17-r1188 (Li and Durbin 2010) was used to map the MGI data, and minimap2 v2.21-r1071 (Pertea et al. 2016) was used to map the HiFi data to genome to calculate the mapping rate.

**Orthologous gene family identification, phylogenetic analysis, estimation of divergence time, and expansion and contraction of gene family expansion**

By using OrthoFinder v2.5.2 (https://github.com/davidemms/OrthoFinder), we obtained orthogroups for 15 species (Supplementary Table 5), and the protein sequences of 217 single-copy orthogroups were obtained. The MAFFT v7.490 software with default settings (Katoh and Standley 2013) was used to perform sequence alignments for each single-copy gene family and the alignments were converted to a nucleotide matrix by pal2nal v14 (Suyama et al. 2006). Under the GTRGAMMA model, a phylogenetic tree was constructed using RAxML v8.2.12 (Stamatakis 2014). Species divergence time was calculated using the MCMCTree program of the PAML v4.9 package (Yang 2007) and treePL v2.6.3 (Smith and O'Meara 2012), based on three soft bounds at three nodes (Cheng et al. 2021b). The calibration points were the 115-308 mya between *Vitis vinifera* and *Zostera marina*, the 125-141 mya between *Z. marina* and *Oryza sativa*, and the 100-118 mya between *Daemonorops jenkinsiana* and *Ensete ventricosum* from the TimeTree database (http://www.time.org/).

Based on the dated phylogeny, we performed expansion and contraction analyses of homologous gene families in the 15 species using the CAFE v4.21 program (Bie et al. 2006). GO and KEGG enrichment analyses were then performed with genes in significantly expanded families.

**Duplicated gene identification and WGD analysis**

To study the size evolution of the *C. alismatifolia* genome, we identified whole genome duplication events in *C. alismatifolia*. We employed DupGen_finder (Qiao et al. 2019) since it integrates the results of MCScanX (Wang et al. 2012) and can identify other classes of duplicated genes. We identified five types of duplicated genes in *C. alismatifolia*, *Z officinale*, and *M. acuminata*, including dispersed duplicates (DSD), proximal duplicates (PD), tandem duplicates (TD), transposed duplicates (TRD), and whole genome duplicates (WGD) by utilizing the DupGen_finder (Qiao et al. 2019) software with default parameters. WGD inference was based on the *Ks* distribution and fitted *Ks* distribution in the collinear blocks of *C. alismatifolia* paralogs, *Z. officinale* paralogs, and orthologs between *C. alismatifolia* and *Z. officinale* obtained by DupGen_finder (Qiao et al. 2019). WGDI (<https://github.com/SunPengChuan/wgdi>) was used to verify the WGD results. The program JCVI v1.1.18 (Tang et al. 2008) was used to further analyze the collinearity of *C. alismatifolia* and *Z. officinale*. In order to verify the accuracy of the *Ks* method, the Tree2gd v1.0.39 software (https://github.com/Dee-chen/Tree2gd), a phylogenetic method (Zhang et al. 2020; Zhao et al. 2021) based on trees constructed from single-copy genes of 15 species was used to detect the whole genome duplication with the default parameters. The WGD determination criteria for the use of the method was described in previous studies (Wang et al. 2021; Zhang et al. 2020). Basically, a whole genome duplication event is considered to have occurred if any of the following conditions are met: (1) gene duplication (GD) events > 500, of which (AB) (AB) type duplication events > 250; (2) gene duplication (GD) events > 1,500, of which (AB)(AB) type duplication events > 100, and the sum of (AB)(AB) type duplication events and (AB)A type or (AB)B type duplication events > 1,000. The KaKs_Calculator and calculate_4DTV_correction.pl integrated in Tree2gd were also used to further calculate the *Ka*, *Ks*, *Ka*/*Ks* and substitution rate of 4dtv sites of gene pairs in the above-mentioned collinear blocks and five types of duplicated genes.

**Methylation analysis**

Genomic DNA (2μg) was obtained for ONT (Oxford Nanopore Technology) library preparations. The ONT PromethION sequencer was used to perform single-molecule sequencing of DNA strands, which produced 97.78 Gb of Nanopore data. The number of reads was 4,636,747, the average read length was 21.09 kb, the reads N50 was 27.20 kb, and the longest read length was 153.92 kb. The call-methylation module of Nanopolish v0.13.2 (https://github.com/jts/nanopolish) was used to analyze 5-methylcytosine in the CG context in the genome based on Fast5 files, then the results were filtered according to the condition of methylated_frequency ≥ 0.5.

**Bulked segregant analysis**

The *C. alismatifolia* ‘Scarlet’ (JL*,* red line) and *C. alismatifolia* ‘Dawn’ (LM, pink line) used for BSA were planted in the Environmental Horticulture Research Institute, Guangdong Academy of Agricultural Sciences. The individual plants of JL and LM grown under natural conditions were used for crossbreeding to obtain F1 hybrid populations. The segregation ratio was 502:483. We extracted DNA and RNA from parental LM (P1) and JL (P2), F1 hybrids LM (50 individuals mixed, S1) and JL (50 individuals mixed, S2), and then sequenced these samples on an Illumina NovaSeq 6000 platform. The DNA and RNA data of P1 was 22.04 Gb and 8.74 Gb, respectively; the DNA and RNA data of P2 was 24.14 Gb and 8.58 Gb, respectively; the DNA and RNA data of S1 was 60.43 Gb and 21.2 Gb, respectively; the DNA and RNA data of S2 was 63.34 Gb and 22.65 Gb, respectively. Data filtering and quality control were performed by Fastp v0.19.4 (Chen et al. 2018). BWA v0.7.17-r1188 (Li and Durbin 2010) was used to map DNA data to the genome, STAR v2.7.9a (Dobin et al. 2013) was used to map RNA data to the genome, then GATK v4.2.2.0 (DePristo et al. 2011) was used for SNP calling. Finally, BSA and BSR analysis was performed using the R package QTLseqr v0.7.5.2 (Mansfeld and Grumet 2018). Hisat2 v2.2.1 (Kim et al. 2019) was used to align the clean reads of RNA-seq from each sample to the *C. alismatifolia* genome. StringTie v2.1.6 (Pertea et al. 2016) was also used to calculate the Fpkm. At the same time FeatureCounts v2.0.1 (Liao et al. 2014) was used to calculate the counts of each gene in each sample, and the R package edgeR v3.36.0 (Robinson et al. 2010) was used to analyze the differentially expressed genes of S2 vs S1 and P2 vs P1, using the following FC value range as the criteria for selecting DEGs: |logFC| ≥ 1, FDR ≤ 0.01.

**Gene co-expression networks**

Based on the Fpkm of all genes, the co-expression network was constructed using the R package WGCNA v1.70-3 (Langfelder and Horvath 2008). By calculating the scale-free topology fit index, the function pickSoftThreshold provided an appropriate soft-thresholding power (β = 12 in this study) for network construction. The minimum module size was set to 30, and modules with highly correlated eigengenes (the cut-off was 0.25) were merged. Combined with the phenotypic information of bract color, the correlation between gene module and phenotype was calculated, and the module most related to green bracts was identified. The relationship between genes and modules was measured by calculating the KME value (module eigengene-based connectivity) to screen the hub genes in the module, where hub genes are those genes that show most connections in the network as indicated by their high KME (eigengene connectivity) values. Here we selected 50 genes with high |KME| value as hub genes.

**Volatile compounds analysis**

A solid-phase microextraction method was used to collect volatile compounds from *C. alismatifolia* “Chiang Mai Pink” in full bloom. Blooming florets samples were added to 15-mL headspace bottle, weighed and balanced for extraction, and the extracted fiber was immediately inserted into the GC injection port for thermal analysis. Analysis was performed using a GCMS-QP2010 system (Shimadzu, Japan). The ion source temperature was set to 200°C and the interface temperature was set to 250°C to get the chromatograms and mass spectrograms of the floral compounds. These compounds were then qualitatively analyzed by the NIST2011 library and compared with published plant volatiles in The Pherobase Database (www.pherobase.com).

**Resequencing and population analysis**

BWA v0.7.17-r1188 (Li and Durbin 2010) was used to map resequencing data of 56 *C. alismatifolia* cultivars to the genome, then GATK v4.2.2.0 (DePristo et al. 2011) was used for SNP calling. PCA was performed using the PLINK v2.00a2.3LM (Chang et al. 2015) software on the filtered 3,706,809 variants. The top three principal components were used for assigning the 56 accessions and downstream population structure analysis. Ancestral population stratification among 56 accessions was inferred using Admixture v1.3.0 (Alexander et al. 2009) software. The red and non-red populations were used to calculate the *Fst* using VCFtools v0.1.16 (Danecek et al. 2011) with a 5kb window size and the mean *Fst* of 0.04 was used as the cut-off. VCF2Dis v1.46 (https://github.com/hewm2008/VCF2Dis) was used to construct phylogenetic tree based VCF file.

**Other analysis**

The iTAK v1.7 program (Zheng et al. 2016) was used to predict transcription factors of *C. alismatifolia*, and the R package corrplot v0.92 (https://github.com/taiyun/corrplot) was used to perform correlation coefficient analyses between genes. The MISA perl script (Thiel et al. 2003) was used for Simple Sequence Repeat (SSR) characterization and most of results were visualized by utilizing the Hiplot web site (https://hiplot.com.cn/basic). The Blast+ v2.11.0 (Altschul et al. 1990) software was used to search the candidates based on the MYB reference of *Arabidopsis thaliana* listed in Dubos *et al*., 2010 (Dubos et al. 2010), bHLH listed in Heim et al. 2003 and Bailey *et al.*, 2003 (Bailey et al. 2003; Heim et al. 2003). The sequence alignment were generated using MAFFT v7.464 (Katoh and Standley 2013) software, and TrimAL v1.4 (Capella-Gutierrez et al. 2009) software was used to trim the poorly aligned positions, then IQ-TREE v2.0 (Minh et al. 2020) was used to reconstruct the phylogenetic tree separately with 1000 ultrafast bootstrap replicates to assess branch support with FigTree v1.4.3 (http://tree.bio.ed.ac.uk/software/figtree) used for tree visualization.

**References**

Alexander DH, Novembre J, Lange K (2009) Fast model-based estimation of ancestry in unrelated individuals. Genome Res 19:1655-1664. <http://dx.doi.org/10.1101/gr.094052.109>

Altschul SF, Gish W, Miller W et al (1990) Basic local alignment search tool. J Mol Biol 215:403-410. <http://dx.doi.org/10.1016/S0022-2836(05)80360-2>

Bailey PC, Martin C, Toledo-Ortiz G et al (2003) Update on the basic helix-loop-helix transcription factor gene family in Arabidopsis thaliana. Plant Cell 15:2497-2502. <http://dx.doi.org/10.1105/tpc.151140>

Bie TD, Cristianini N, Demuth JP et al (2006) CAFE: a computational tool for the study of gene family evolution. Bioinformatics 22:1269-1271. http://dx.doi.org/10.1093/bioinformatics/btl097

Burton JN, Adey A, Patwardhan RP et al (2013) Chromosome-scale scaffolding of de novo genome assemblies based on chromatin interactions. Nat Biotechnol 31:1119-1125. <http://dx.doi.org/10.1038/nbt.2727>

Capella-Gutierrez S, Silla-Martinez JM, Gabaldon T (2009) trimAl: a tool for automated alignment trimming in large-scale phylogenetic analyses. Bioinformatics 25:1972-1973.

Chang CC, Chow CC, Tellier LC et al (2015) Second-generation PLINK: rising to the challenge of larger and richer datasets. Gigascience 4:7. <http://dx.doi.org/10.1186/s13742-015-0047-8>

Chen SF, Zhou YQ, Chen YR et al (2018) fastp: an ultra-fast all-in-one FASTQ preprocessor. Bioinformatics 34:884-890. <http://dx.doi.org/10.1093/bioinformatics/bty560>

Cheng HY, Concepcion GT, Feng XW et al (2021a) Haplotype-resolved de novo assembly using phased assembly graphs with hifiasm. Nat Methods 18:170-175. <http://dx.doi.org/10.1038/s41592-020-01056-5>

Cheng SP, Jia KH, Liu H et al (2021b) Haplotype-resolved genome assembly and allele-specific gene expression in cultivated ginger. Hortic Res 8:188. http://dx.doi.org/10.1038/s41438-021-00599-8

Danecek P, Auton A, Abecasis G et al (2011) The variant call format and VCFtools. Bioinformatics 27:2156-2158. <http://dx.doi.org/10.1093/bioinformatics/btr330>

DePristo MA, Banks E, Poplin R et al (2011) A framework for variation discovery and genotyping using next-generation DNA sequencing data. Nat Genet 43:491-498. http://dx.doi.org/10.1038/ng.806

Dobin A, Davis CA, Schlesinger F et al (2013) STAR: ultrafast universal RNA-seq aligner. Bioinformatics 29:15-21. <http://dx.doi.org/10.1093/bioinformatics/bts635>

Dubos C, Stracke R, Grotewold E et al (2010) MYB transcription factors in Arabidopsis. Trends in Plant Science 15:573-581. <http://dx.doi.org/10.1016/j.tplants.2010.06.005>

Heim MA, Jakoby M, Werber M et al (2003) The basic helix-loop-helix transcription factor family in plants: a genome-wide study of protein structure and functional diversity. Mol Biol Evol 20:735-747. <http://dx.doi.org/10.1093/molbev/msg088>

Hu J, Fan JP, Sun ZY et al (2020) NextPolish: a fast and efficient genome polishing tool for long-read assembly. Bioinformatics 36:2253-2255. <http://dx.doi.org/10.1093/bioinformatics/btz891>

Katoh K, Standley DM (2013) MAFFT multiple sequence alignment software version 7: improvements in performance and usability. Mol Biol Evol 30:772-780. <http://dx.doi.org/10.1093/molbev/mst010>

Kim D, Paggi JM, Park C et al (2019) Graph-based genome alignment and genotyping with HISAT2 and HISAT-genotype. Nat Biotechnol 37:907-915. <http://dx.doi.org/10.1038/s41587-019-0201-4>

Langfelder P, Horvath S (2008) WGCNA: an R package for weighted correlation network analysis. BMC Bioinform 9:559. http://dx.doi.org/10.1186/1471-2105-9-559

Langmead B, Salzberg SL (2012) Fast gapped-read alignment with Bowtie 2. Nat Methods 9:357-359. <http://dx.doi.org/10.1038/Nmeth.1923>

Li H, Durbin R (2010) Fast and accurate long-read alignment with Burrows-Wheeler transform. Bioinformatics 26:589-595. <http://dx.doi.org/10.1093/bioinformatics/btp698>

Liao Y, Smyth GK, Shi W (2014) featureCounts: an efficient general purpose program for assigning sequence reads to genomic features. Bioinformatics 30:923-930. <http://dx.doi.org/10.1093/bioinformatics/btt656>

Liu BH, Shi YJ, Yuan JY et al (2013) Estimation of genomic characteristics by analyzing k-mer frequency in de novo genome projects. arXiv:1308.2012. https://doi.org/10.48550/arXiv.1308.2012

Mansfeld BN, Grumet R (2018) QTLseqr: an R package for bulk segregant analysis with next-generation sequencing. Plant Genome 11:1-5. <http://dx.doi.org/10.3835/plantgenome2018.01.0006>

Marcais G, Kingsford C (2011) A fast, lock-free approach for efficient parallel counting of occurrences of k-mers. Bioinformatics 27:764-770. <http://dx.doi.org/10.1093/bioinformatics/btr011>

Wang M, Wang T, Xia ZQ et al (2021) Revealing the new whole-genome duplication event of four paphiopedilum species based on transcriptome data. Chinese Bulletin of Botany 56:699-714. <http://dx.doi.org/10.11983/CBB21100>

Minh BQ, Schmidt HA, Chernomor O et al (2020) IQ-TREE 2: New Models and Efficient Methods for Phylogenetic Inference in the Genomic Era. Mol Biol Evol 37:1530-1534.

Parra G, Bradnam K, Korf I (2007) CEGMA: a pipeline to accurately annotate core genes in eukaryotic genomes. Bioinformatics 23:1061-1067. <http://dx.doi.org/10.1093/bioinformatics/btm071>

Pertea M, Kim D, Pertea GM et al (2016) Transcript-level expression analysis of RNA-seq experiments with HISAT, StringTie and Ballgown. Nat Protoc 11:1650-1667. http://dx.doi.org/10.1038/nprot.2016.095

Qiao X, Li QH, Yin H et al (2019) Gene duplication and evolution in recurring polyploidization-diploidization cycles in plants. Genome Biol 20:38. http://dx.doi.org/10.1186/s13059-019-1650-2

Robinson MD, McCarthy DJ, Smyth GK (2010) edgeR: a Bioconductor package for differential expression analysis of digital gene expression data. Bioinformatics 26:139-140. <http://dx.doi.org/10.1093/bioinformatics/btp616>

Servant N, Varoquaux N, Lajoie BR et al (2015) HiC-Pro: an optimized and flexible pipeline for Hi-C data processing. Genome Biol 16:259 http://dx.doi.org/10.1186/s13059-015-0831-x

Simao FA, Waterhouse RM, Ioannidis P et al (2015) BUSCO: assessing genome assembly and annotation completeness with single-copy orthologs. Bioinformatics 31:3210-3212. <http://dx.doi.org/10.1093/bioinformatics/btv351>

Smith SA, O'Meara BC (2012) treePL: divergence time estimation using penalized likelihood for large phylogenies. Bioinformatics 28:2689-2690. <http://dx.doi.org/10.1093/bioinformatics/bts492>

Stamatakis A (2014) RAxML version 8: a tool for phylogenetic analysis and post-analysis of large phylogenies. Bioinformatics 30:1312-1313. <http://dx.doi.org/10.1093/bioinformatics/btu033>

Suyama M, Torrents D, Bork P (2006) PAL2NAL: robust conversion of protein sequence alignments into the corresponding codon alignments. Nucleic Acids Res 34:609-612. http://dx.doi.org/10.1093/nar/gkl315

Tang HB, Bowers JE, Wang XY et al (2008) Synteny and collinearity in plant genomes. Science 320:486-488. <http://dx.doi.org/10.1126/science.1153917>

Thiel T, Michalek W, Varshney RK et al (2003) Exploiting EST databases for the development and characterization of gene-derived SSR-markers in barley (Hordeum vulgare L.). Theor Appl Genet 106:411-422. <http://dx.doi.org/10.1007/s00122-002-1031-0>

Wang Y, Tang H, Debarry JD et al (2012) MCScanX: a toolkit for detection and evolutionary analysis of gene synteny and collinearity. Nucleic Acids Res 40:e49. <http://dx.doi.org/10.1093/nar/gkr1293>

Yang ZH (2007) PAML 4: Phylogenetic analysis by maximum likelihood. Molecular Biology and Evolution 24:1586-1591. <http://dx.doi.org/10.1093/molbev/msm088>

Zhang CF, Zhang TK, Luebert F et al (2020) Asterid phylogenomics/phylotranscriptomics uncover morphological evolutionary histories and support phylogenetic placement for numerous whole-genome duplications. Mol Biol Evol 37:3188-3210. <http://dx.doi.org/10.1093/molbev/msaa160>

Zhao YY, Zhang R, Jiang KW et al (2021) Nuclear phylotranscriptomics and phylogenomics support numerous polyploidization events and hypotheses for the evolution of rhizobial nitrogen-fixing symbiosis in Fabaceae. Mol Plant 14:748-773. <http://dx.doi.org/10.1016/j.molp.2021.02.006>

Zheng Y, Jiao C, Sun H et al (2016) iTAK: a program for genome-wide prediction and classification of plant transcription factors, transcriptional regulators, and protein kinases. Mol Plant 9:1667-1670. <http://dx.doi.org/10.1016/j.molp.2016.09.014>
